# A preliminary analysis on the effect of copper on *Anopheles coluzzii* insecticide resistance in vegetable farms in Benin

**DOI:** 10.1038/s41598-020-63086-5

**Published:** 2020-04-14

**Authors:** Armand Defo Talom, Michele Agnes Essoung, Adam Gbankoto, Genevieve Tchigossou, Romaric Akoton, Bio Bangana A. Sahabi, Seun Michael Atoyebi, Apollin Fotso Kuate, Rudi L. Verspoor, Manuele Tamò, Timoleon Tchuinkam, Gustave Leopold Lehman, Jo Lines, Charles S. Wondji, Rousseau Djouaka

**Affiliations:** 1 University of Dschang, Vector Borne Diseases Laboratory (VBID), Po Box 067, Dschang, Cameroon; 2 International Institute of Tropical Agriculture, Yaoundé, Cameroon; 3 University of Abomey Calavi, Laboratory of Experimental Physiology and Pharmacology, Faculty of Sciences and Technology BP 526, Cotonou, Benin; 4 International Institute of Tropical Agriculture, Cotonou, 08 BP 0932 Benin; 5 National University of Agriculture, Porto-Novo, Benin; 6 Cell Biology and Genetics Unit, Department of Zoology, University of Ibadan, P.O. Box 5116, Oyo State, Nigeria; 7 University of Liverpool, Institute of Integrative Biology, Liverpool, L697ZB United Kingdom; 8 Faculty of Science University of Douala, Douala, Cameroon; 9 London School of Hygiene & Tropical Medicine, London, UK; 10 Liverpool School of Tropical Medicine, Pembroke Place, L3 5QA, Liverpool, UK; 11 Centre for Research in Infectious Diseases (CRID), Yaoundé, Cameroon

**Keywords:** Molecular biology, Malaria

## Abstract

The use of agrochemicals in vegetable production could influence the selection for insecticide resistance in malaria vectors. Unfortunately, there is a dearth of information on the potential contribution of agrochemicals to insecticide resistance in *Anopheles* mosquitoes breeding on vegetable farms in southern Benin. A Knowledge, Attitudes and Practices study was conducted with 75 vegetable farmers from Houeyiho and Seme to determine the main agrochemicals used in vegetable production, and the concentration and frequency of application, among other details. Mosquitoes and breeding water were sampled from the farms for analysis. Bioassays were conducted on mosquitoes, while breeding water was screened for heavy metal and pesticide residue contamination. Lambda-cyhalothrin was the main insecticide (97.5%) used by farmers, and *Anopheles coluzzii* was the main mosquito identified. This mosquito species was resistant (30–63% mortality rate) to λ-cyhalothrin. It was also observed that 16.7% of the examined breeding sites were contaminated with λ-cyhalothrin residues. Furthermore, copper contamination detected in mosquito breeding sites showed a positive correlation (r = 0.81; P = 0.0017) with mosquito resistance to λ-cyhalothrin. The presence of copper in λ-cyhalothrin-free breeding sites, where mosquitoes have developed resistance to λ-cyhalothrin, suggests the involvement of copper in the insecticide resistance of malaria vectors; this, however, needs further investigation.

## Introduction

Integrated vector control strategies, such as larval control, indoor residual spraying (IRS) and the use of long-lasting insecticide-treated nets (LLITNs), rely mainly on the use of synthetic products such as chemical insecticides, which have been used for decades in public health and agriculture. Africa has approximately 874 million hectares of arable land which, while underutilised, the continent still remains an active global producer of foods^1^. In recent times, there has been improved awareness and redirection to agriculture across Africa, which has led to increased agricultural activities on the continent. Presently, the agricultural sector accounts for 60% of employment in Africa, so in an attempt to improve crop yields to meet wage demands and increase profits, farmers engage in the continuous use and overuse of pesticides and chemical fertilisers^2–5^. Poor farming practices, including the misuse of pesticides, have been reported to enhance environmental selection for pesticide resistance^6–8^. Apart from its effect on target organisms, agrochemical use in agriculture also interferes with other nontarget insects, some of which are of public health importance (mosquitoes)^9–12^.

There are different classes of insecticides that have been used since the 1950s, but current control programmes depend mainly on synthetic pyrethroids^13^. The effectiveness of pyrethroid insecticides against malaria vectors has been well investigated, and the resulting findings have been crucial for policy designed to reduce malaria transmission in endemic areas^14,15^. However, reports of increasing and spreading pyrethroid resistance are weakening the expected success of the use of this chemical compound for malaria control^16–18^. Resistance has been associated with the increased use of these synthetic products in public health interventions^19–22^. Other reports have also linked mosquito exposure to agrochemicals as a contributing factor to pyrethroid resistance in mosquito populations^8,23–25^.

Pyrethroid pesticides such as deltamethrin, λ-cyhalothrin and permethrin are simultaneously used for agricultural pest management and vector control^26,27^. There are a few observations in the literature that have been able to associate the use of insecticides in agriculture with cross-resistance in disease vectors^28^. The general theory behind this phenomenon is that during irrigation/rainfall, insecticide residues generated from agricultural treatments are washed into mosquito breeding sites, thus exerting selection pressure on larval populations^12,29^. The risk of this cross-resistance, however, could increase if the agrochemicals have similar active ingredients or modes of action in vectors and in pest insects. In addition, some non-insecticidal chemicals, such as fungicides and herbicides, as well as compounds, such as heavy metals, may also contribute to resistance selection in disease vectors^9,30,31^.

The swampy and wet lands used for agriculture are good breeding sites for agricultural pests and mosquito species and thus may constitute a threat to malaria vector control if there is an accumulation of agrochemical residues in breeding sites or abuse of agrochemicals used for pest control^10,28,32^. A clear understanding of the effect of agrochemical use in agriculture on the spread of insecticide resistance in disease vectors is hereby required. In Benin, there are reports that a variety of synthetic agrochemicals are used for pest control in vegetable farms^33^. Some of these agrochemicals are registered, while some are not^12,29,33^.

In this study, however, we have presented information on the common insecticides used by vegetable farmers in southern Benin and have analysed the potential contribution of copper residues from fertilisers to the selection for resistance in malaria vectors.

## Results

### Micro-mapping of breeding sites

**Figure 1:**
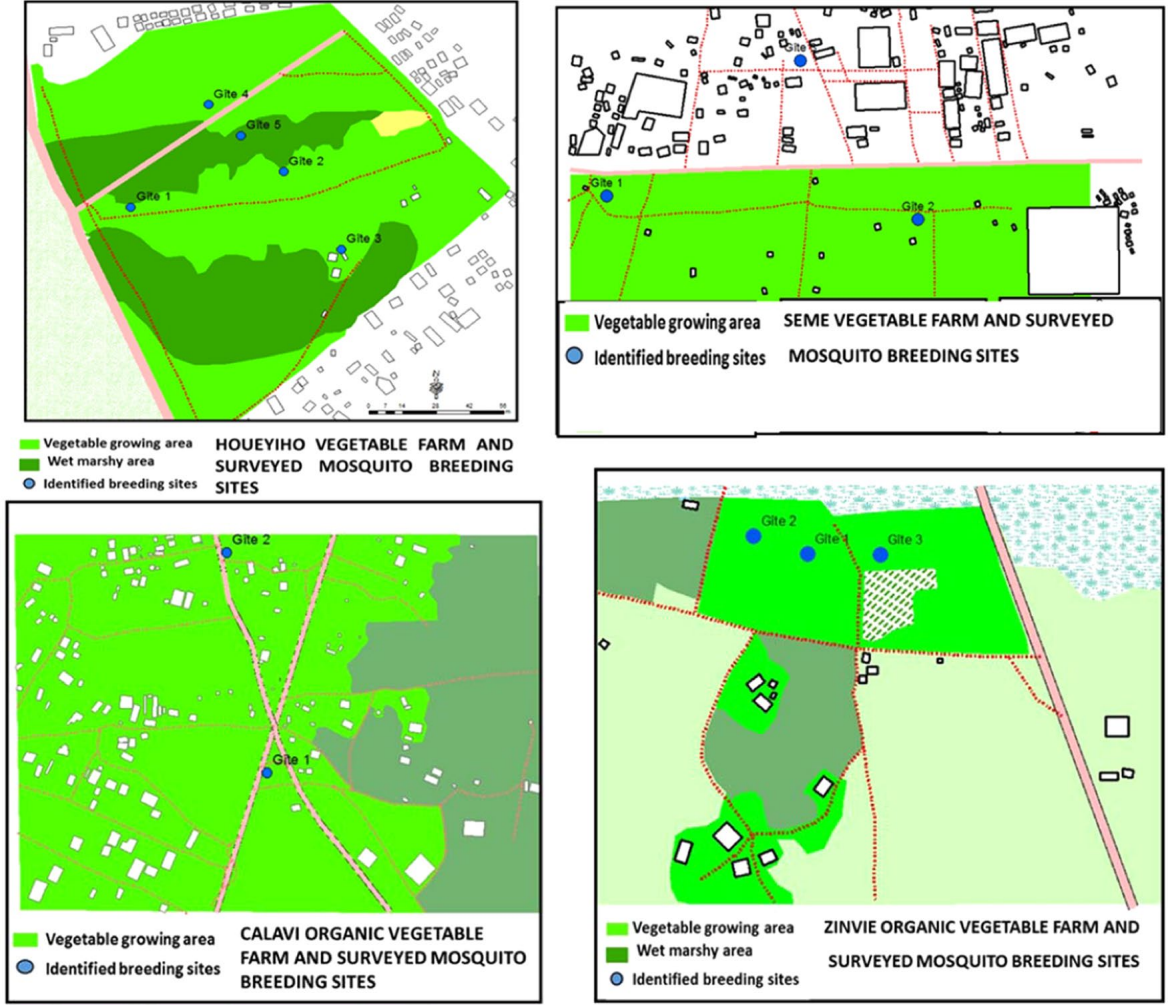
Maps of mosquito breeding sites: (**a**) Houeyiho farm under synthetic pesticide treatments; (**b**) Seme farm under synthetic pesticide treatments; (**c**) Calavi organic farm, no chemical pesticide utilisation; (**d**) Seme organic farm, no chemical pesticide utilisation.

*Anopheles* mosquito larvae were collected from a total of twelve breeding sites on four (4) different vegetable farms (Fig. 1a–d). Eight of these breeding sites were identified on test farms where λ-cyhalothrin insecticide was used (five in Houeyiho and three in Seme), while four were on control farms where no synthetic insecticide was used (two in Calavi and two in Zinvie). The unbalanced number of breeding sites was due to the locations of the surveyed sites (low/wetlands and uplands), the amounts of water used and the types of irrigation systems. The identified breeding sites at Houeyiho were at a distance of 30 m away from one another, while this distance at Seme was approximately 50 m.

Unlike Houeyiho, which is located in the lowland area of Cotonou and wet throughout the year, the other three vegetable farms (Seme, Calavi and Zinvie) experience some level of drought during the dry season, which farmers address with irrigation through the use of water storage systems such as tanks and large containers. We noticed that some agrochemicals (pesticides) were applied to plants by incorporation of these substances into the irrigation water. It was observed that the cans used for irrigation also served as the most common site for mosquito larvae development during dry seasons.

### Agrochemical usage in vegetable farms

**Figure 2:**
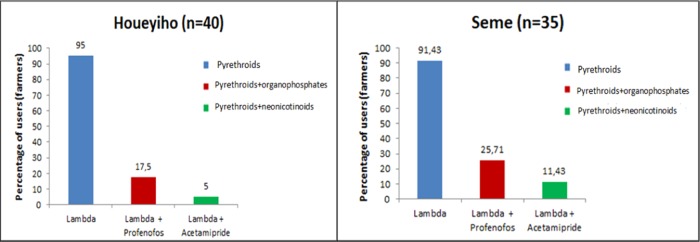
Main insecticides used for vegetable production at the Houeyiho (n = 40) and Seme (n = 35) vegetable farms.

From the KAP study, information on vegetable production and the use of synthetic pesticides during vegetable cultivation was obtained from a total of 75 farmers (40 from Houeyiho and 35 from Seme), which included only 3 women as the profession is mainly dominated by men. This survey revealed that farmers grow different types of vegetables, such as lettuce, cabbage, spinach, cucumber, chili pepper, and pepper, throughout the year. The active ingredient in the insecticide used by most farmers (97.5%) is λ-cyhalothrin, which is used by 95 and 91.4% of farmers in Houeyiho and Seme, respectively. The next most common insecticide used is the combination of λ-cyhalothrin (a pyrethroid) and profenofos (an organophosphate), which is used by 17.5% and 25.7% of farmers in Houeyiho and Seme, respectively (Fig. 2a,b).

Approximately 52.5 and 68.6% of farmers in Houeyiho and Seme, respectively, apply λ-cyhalothrin twice a month (14-day treatment interval), mainly on lettuce (Table 1). However, 30 and 20% of farmers in Houeyiho and Seme, respectively, prefer to apply λ-cyhalothrin every 7 days (4 treatments/ month). For insecticide dilution, during each application, we noticed that only 57.5 and 62.9% of farmers in Houeyiho and Seme, respectively, adhered strictly to the manufacturer’s standard protocol of diluting 5 mL of λ-cyhalothrin in 10 L of water (Table 1)

**Table 1:** The frequency of application and dilution of insecticides used by farmers on Houeyiho and Seme vegetable farms.

Insecticide usage on surveyed vegetable farms		Houeyiho		Seme	
		n	%	n	%
Frequency of insecticide treatment	Every 7 days	12	30	7	20,0
	Every 10 days	3	7,5	2	5,7
	Every 14 days	21	52,5	24	68,6
	Every 30 days	4	10	2	5,7
Insecticide dilution	2.5 mL/10 L	14	35	11	31,4
	3 mL/10 L	3	7,5	2	5,7
	5 mL/10 L	23	57,5	22	62,9
Total respondents	40	100	35	100	

### Insecticide susceptibility test

PCR revealed that all female (n = 400) *An. gambiae sl*. (100 *An. gambiae sl*. per vegetable farm) analysed for species identification were *An. coluzzii*.

**Figure 3:**
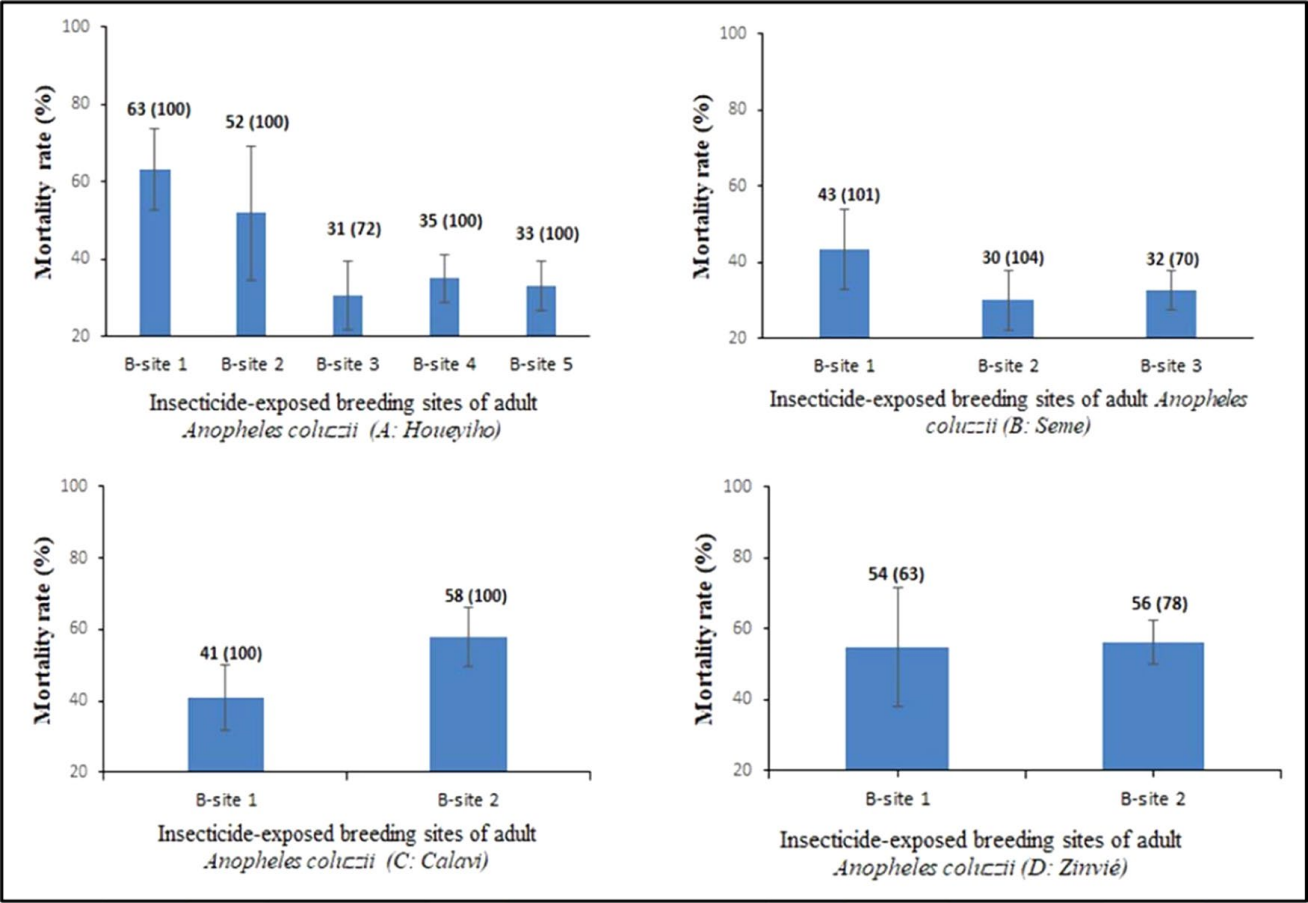
Susceptibility profiles of *An. coluzzii* from conventional vegetable sites of (**a**) Houeyiho, (**b**) Seme, (**c**) Calavi and (**d**) Zinvie to λ-cyhalothrin. Histogram bars represent the mean mortality rates; error bars represent the standard deviations of the mean.

Insecticide susceptibility tests showed that mosquitoes collected from within and across vegetable farms have developed high levels of resistance to λ-cyhalothrin. Mosquito samples from the 5 georeferenced breeding sites at Houeyiho showed a mortality range of 31 to 63% when exposed to λ-cyhalothrin (Fig. 3a). However, at Seme, the mortality rate of mosquito populations from the 3 georeferenced breeding sites was between 30 and 43% (Fig. 3b).

Surprisingly, the mortality rates of mosquitoes from Calavi and Zinvie (control sites), where no synthetic chemical insecticide was used, were also low when exposed to λ-cyhalothrin. At Calavi, the rates were 41 and 58% for the 2 georeferenced breeding sites, and the rates were 54 and 56% for the 2 breeding sites found at Zinvie (Fig. 3a,b,c,d).

### Presence of insecticide residues in georeferenced mosquito breeding sites

**Figure 4:**
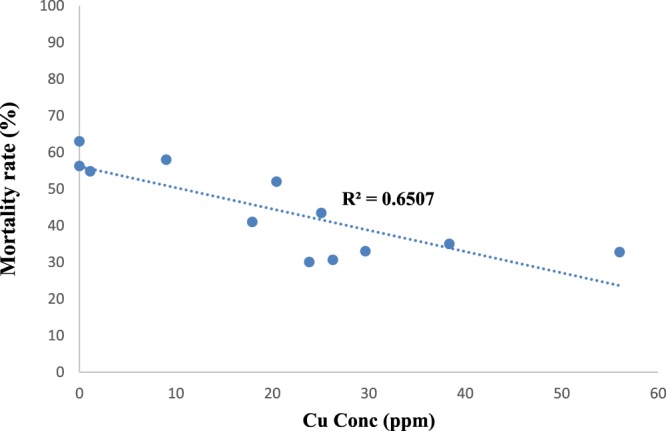
Cross analysis showing high resistance levels (low mortalities) of *An. coluzzi* emerging from breeding sites on the four surveyed vegetable farms with high copper concentrations.

Insecticide residue screening of the 12 georeferenced breeding sites revealed that only two breeding sites (from Houeyiho farm) contained λ-cyhalothrin residues. (Table 2). It was also observed that mosquitoes from these two breeding sites had the highest resistance level, showing 31 and 33% mortality (Fig. 4). Overall, a t-test (using GraphPad Prism 5) revealed that there was a significant relationship (P = 0.0014) between the presence of λ-cyhalothrin residues and the high insecticide resistance of mosquitoes observed in these breeding sites.

**Table 2:** Concentrations of λ-cyhalothrin residues in *Anopheles coluzzii* breeding sites identified in Houeyiho, a farm using synthetic insecticide treatments.

Localities	Houeyiho					Seme			Calavi		Zinvie	
Breeding sites	BS1	BS2	BS3	BS4	BS5	BS1	BS2	BS3	BS1	BS2	BS1	BS2
Conc of λ-Cyhalothrin	ND	ND	0.005 ± 0.008	ND	0.277 ± 0.090	ND	ND	ND	ND	ND	ND	ND

BS: Breeding site; ND: Not detected.

### Possible relationship between the presence of copper in the breeding sites and insecticide resistance profiles of *Anopheles* mosquitoes

Copper was detected in the sampled mosquito breeding sites across the 4 vegetable farms at concentrations that ranged from 0.2043 to 0.5601 ppm. Furthermore, we observed that the breeding sites that were contaminated with Cu also had low mosquito mortalities, indicating high resistance. Cross-analysis, however, revealed that there was a positive correlation (linear regression analysis gave r = 0.81; P = 0.0017) between the presence of Cu in the breeding sites and the resulting λ-cyhalothrin resistance of mosquitoes from the same breeding site (Fig. 4). This observed correlation between the presence of Cu in breeding sites and the increased resistance to λ-cyhalothrin is being further investigated in our laboratory.

### Laboratory monitoring of the effect of Cu on known pyrethroid-susceptible and -resistant mosquito populations

**Figure 5:**
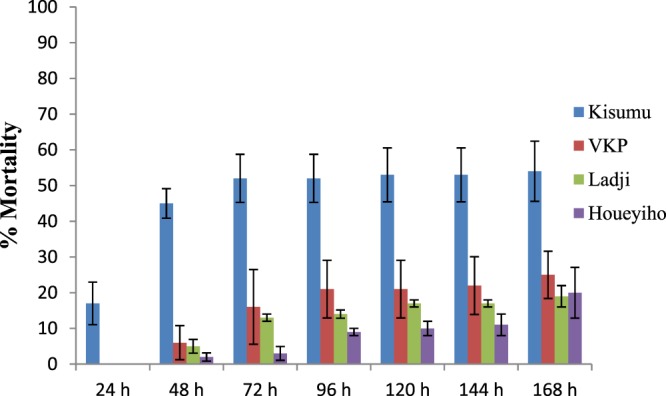
Mortality rates of larvae of resistant field strains (Ladji and Houeyiho), a resistant laboratory strain (VKPer) and a susceptible laboratory strain (Kisumu) bred in media contaminated with copper solutions.

For the breeding site simulation study, the susceptible strain of *Anopheles gambiae Kisumu* had the highest mortality compared to other mosquito strains after 168 hours of exposure (Fig. 5). There was an initial rapid increase in mortality (approximately 52%) from the first minute of exposure until 72 hours after exposure. Then mortality became stable and only slightly increased again after 120 hours of exposure. However, for the known resistant strains (*Anopheles gambiae* VKPER, *Anopheles coluzzii* Ladji and *Anopheles coluzzii* Houeyiho) that were exposed to Cu, we started observing mortalities at 48 hours postexposure and even at 96 hours postexposure, *An. gambiae* VKPER, *An. coluzzii*-Ladji and *An. coluzzii-*Houeyiho only showed 21, 14 and 9% mortalities, respectively.

As expected, resistant populations had lower mortality than *An. gambiae Kisumu*, suggesting a higher level of Cu tolerance in the resistant strains. The trend of Cu tolerance, however, was as follows: *An. gambiae* Ladji > *An. gambiae* Houeyiho > *An. gambiae* VKPER > *An. gambiae Kisumu* (Fig. 5). Overall, there were significant differences in the observed mortalities between *An. gambiae* Houeyiho and *An. gambiae Kisumu* (r = 0.62, P = 0.0032) as well as *An. gambiae* Ladji (r = 0.87, P = 0.0085) and *An. gambiae* VKPER (r = 0.87, P = 0.0104) compared to *An. gambiae Kisumu*. Additionally, there were significant correlations between the different resistant strains (using GraphPad Prism 5, correlation analysis between *An. gambiae* Houeyiho and *An. gambiae* Ladj gave r = 0.84, P = 0.0173; *An. gambiae* Houeyiho and *An. gambiae* VKPER gave r = 0.86, P = 0.0142; *An. gambiae* VKPER and *An. gambiae* Ladji gave r = 0.99, P = 0.0001).

## Discussion

Vegetable production is a fast-growing agricultural activity in urban areas across Benin, and the use of synthetic pesticides remains the principal pest control strategy on these vegetable farms. Unfortunately, the resistance of vegetable pests to insecticides is rapidly growing and may jeopardise the efficacy of these synthetic products^34^. The observed resistance coupled with other information gathered in this study highlights the fact that most of the farmers do not use these insecticides properly during agricultural applications, which may have impacted the level of insecticide resistance of mosquitoes. From observations made during our surveys at Houeyiho and Seme (the two vegetable production sites under the synthetic pesticide regime), we realised that some farmers have neglected standard practices and now formulate their own insecticide concentrations as well as alter the standard frequency of application (14-day intervals between insecticide applications). The impacts of this misuse and overuse of insecticides on the environment and more specifically on non-target organisms such as malaria vectors^8,11,28^ that breed in and around these treated vegetable farms have been emphasised. This poor agricultural practice increases the environmental levels of these chemical metabolites, and their continuous interactions with the environment, including breeding waters, may have a negative effect on the behavioural and physiological state of the organisms utilizing these waters^11,34^. Furthermore, the leaching of pyrethroid insecticides and other agro-chemicals used by farmers into the surrounding breeding water during/after applications^35^ is believed to have an effect on resistance selections in mosquitoes^28,36^. This could have contributed to the observed insecticide resistance profiles. Although the use of pesticides in agriculture has constantly been implicated as a contributing factor to insecticide resistance selection in malaria vectors, this has not been thoroughly investigated in different cropping systems. Therefore, we believe that this knowledge gap should be filled considering the continuous threat of insecticide resistance to malaria vector control programmes in Africa^37–39^. There is, however, a clear understanding that these agro-insecticides are similar, both in their structures and mode of action to those used in public health for controlling disease vectors, which is another reason for examining the interactive effects of insecticides from different sources. There is a belief that these similarities may initiate some unknown interactions between insecticides from different sources, which may have a particularly large impact on disease vectors. A good example of this is a possible interaction between deltamethrin insecticides (a type 2 pyrethroid) used for controlling adult mosquitoes in urban residential areas^40^, and lambda-cyhalothrin insecticide, another type 2 pyrethroid used in vegetable farms around these areas^41^. Therefore, there is every possibility that *An. coluzzii* larvae collected from contaminated sites have interacted and been exposed to different insecticide residues and other resident contaminants (heavy metals such as copper) in their breeding sites, thereby resulting in higher insecticide resistance profiles, as recorded in this study^42^.

The high resistance profiles observed for the organic farm sites showed that mosquito resistance is not restricted to farms that utilize synthetic insecticides. Although synthetic insecticides are not used in organic farming, it is possible that resident mosquitoes might have migrated into the farm for breeding. Additionally, it may be that resistant genes have been inherited and maintained over several generations of the mosquito population or as part of the insecticide residual effect on the mosquito population. The absence of λ-cyhalothrin residue after vegetable treatments in over 80% of the breeding sites suggests the role of other forms of xenobiotics, such as heavy metals, in the selection of insecticide resistance in mosquito populations^28,43^. Vegetable farmers use combinations of different chemical compounds to treat vegetables. These combinations include herbicides, fertilisers and insecticides^9^. The use of a cocktail of chemicals in agriculture has already been raised as contributing to resistance selection in malaria vectors through the continuous exposure of mosquito larvae to different cocktail compositions^9^. Although there is no confirmation that the use of neem oil in organic farms contributes to insecticide resistance selection, some researchers believe that continuous exposure of insects to neem may influence resistance.^44^ Vollinger^45^ showed the absence of resistance to neem in diamond back moth populations after 42 generations. Further investigations are needed to elucidate the contribution of neem to the observed resistance of mosquitoes in organic farms, where synthetic insecticide use is absent. The results from this research, however, highlight that resistance selection is not only from the contact between mosquitoes and LLINs/IRS. Resistance could also result from regular contact between mosquito larvae and insecticidal/non-insecticidal agrochemicals found in their breeding sites during their larval developmental stages. Hence, vegetable farming can be considered an alternative source of insecticide resistance selection, as previously documented by some authors^9,28,46^.

The presence of lambda-cyhalothrin residues in only 2 out of the 12 breeding sites at Houeyiho might be due to its rapid degradation by natural metabolism such as via aquatic organisms residing in breeding sites^47–49^. Another degradation pathway could be exposure to sunlight (photodegradation), which has also been highlighted in the pyrethroid degradation process^41^. Djouaka *et al*.^41^ reported how λ-cyhalothrin degrades rapidly under UV light, especially at the concentrations used by vegetable farmers. Although the degradation of λ-cyhalothrin could be rapid, the little time this insecticide spends in the water can still impact resistance selection and affect larval development over generations.

Apart from the impact of insecticide residue exposure, resistance selection of mosquitoes could also be linked to heavy metal contamination of breeding sites. This was evidenced in this study, as we observed a higher λ-cyhalothrin resistance level with mosquito larvae drawn from breeding sites that were contaminated with Cu. Poupardin *et al*.^30^ and Riaz *et al*.^31^ found that mosquitoes detoxify heavy metals with metabolic enzymes such as cytochrome P450 and glutathione-S-transferase just as they do with insecticides. It is possible that mosquito larvae from vegetable production sites, as they regularly become exposed to cocktails of agrochemicals, have built a cross-resistance system, which allows them to survive Cu and λ-cyhalothrin contaminants found in their breeding environment. The presence of copper in breeding sites may be a result of the decomposition of copper sulfate, a fungicide used by farmers, or of fertilisers generated from chicken and pig droppings, which may contain heavy metals^50^. Vegetable farmers in Houeyiho and Seme use these kinds of fertilisers (derived from animal droppings) without prior treatments; it is therefore possible that copper contents in these droppings leach into mosquito breeding sites during rainfall and make contact with mosquito larvae.

This study further confirms that mosquitoes can survive in metal-contaminated habitats, as previously documented by Mireji *et al*.^43^, and highlights the need for further investigations on the contribution of copper and other non-insecticidal xenobiotics to insecticide resistance selection in malaria vectors.

## Methods

### Study sites

**Figure 6:**
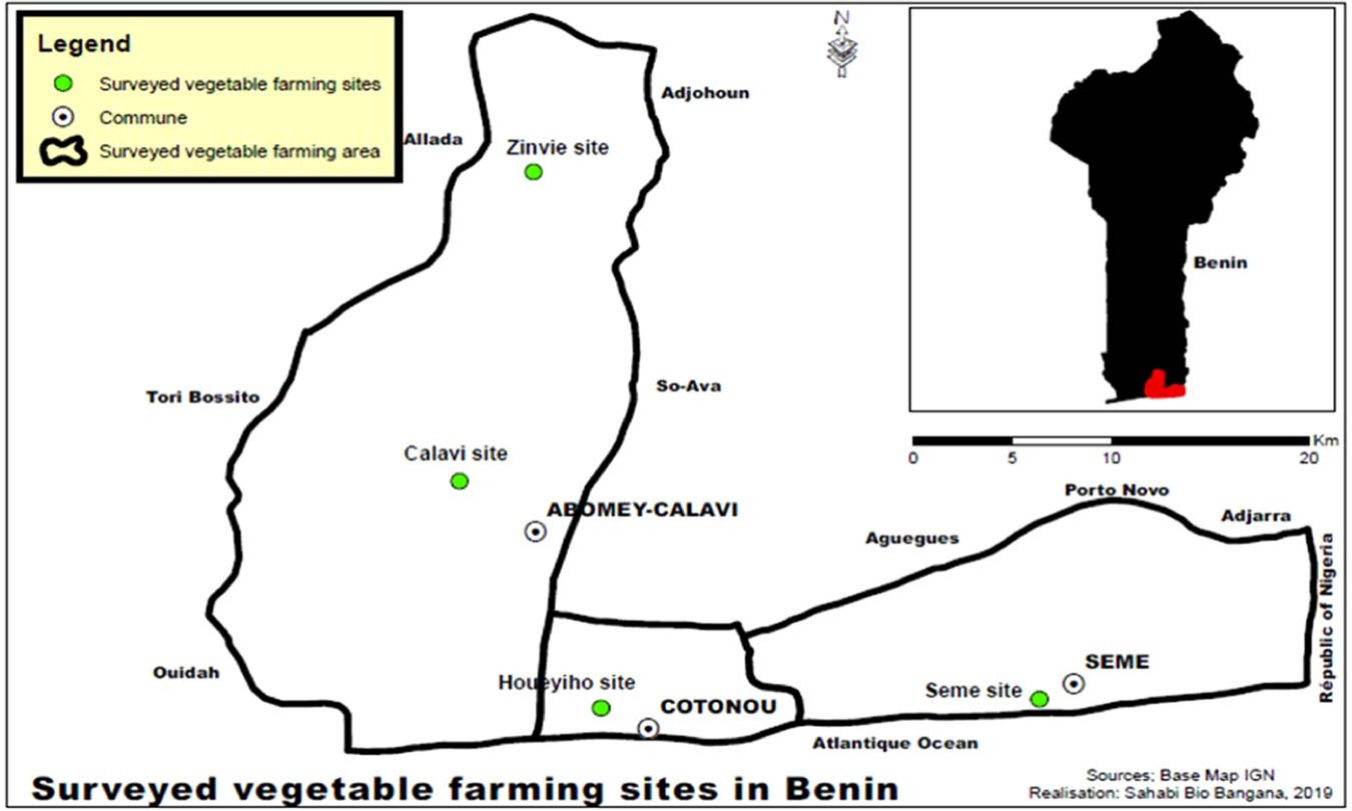
Map of the four surveyed vegetable farms in southern Benin. ArcGIS version 10.4 *software was used to create map (Map Fonts was provided by IGN-Benin (the Benin National Institute of Geography « Institut Geographique National* » http://ign.bj/).

The study was conducted at four vegetable farms (one vegetable farm per locality) in southern Benin (Fig. 6) from January to December 2018 - two non-organic farms where chemical insecticides were used (Houeyiho and Seme) and two organic farms (Calavi and Zinvie) where biological insecticides such as plant extracts were used for pest control (Table 3)

**Table 3:** Description of study sites.

		Location	GPS coodinates	Vegetables produced	Agrochemicals used	Irrigation systems
Non-organic farms	Houeyiho	Cotonou	6°22′0, 2′′N; 2°23′48, 31′′E	cabbages, carrots, lettuces, amaranth and cucumber, etc	insecticides, herbicides, and fungicides and chemical fertilisers	wells, wastewaters, drillings and swamps
	Seme	Seme (approximately 21 km from Cotonou)	6°22′26, 64′′N and 2°34′ 22,1′′E	cabbages, carrots, lettuces, amaranth and cucumber, etc	insecticides, herbicides, and fungicides and chemical fertilisers	wells
Organic farms	Zinvie	Zinvie (approximately 33 km from Cotonou)	6°37′ 0′′N and 2°21′ 0′′E	cabbages, carrots, lettuces	plant extracts (e,g: neem oil)	Borehole
	Calavi	Abomey-Calavi (approximately 14 km from Cotonou)	6°28′ 21,81′′N and 2°19′ 14,38′′E	cabbages, carrots, lettuces	plant extracts (e,g: neem oil)	Borehole

### Knowledge, attitudes and practices (KAPs) of vegetable farmers concerning chemical insecticide usage

A structured questionnaire (supplementary file) was administered in French and a local language to 75 farmers who gave their consent to be part of the study. Information on the use of insecticides was obtained from farmers (men and women) to assess their attitudes, practices, beliefs and understanding of insecticide usage and pest control. The questionnaire also aimed to obtain information on the concentrations, application frequencies, and types of chemical insecticides used. Verbal informed consent was obtained from all participants prior to completing the questionnaire. Ethical clearance for the study was also obtained from the internal Review Board (IRB) of IITA. Information collected from participants at all study sites was treated as private and strictly confidential.

### Collection and rearing of mosquito larvae

An inventory of *Anopheles* breeding sites was first taken, and each identified breeding site was georeferenced prior to mosquito collection. The identified breeding sites included vegetable furrows, swamps, wells, plastic reservoirs and containers, in which irrigation/rain water is mostly stored. *Anopheles spp*. were sampled using the dipping method^51^. Mosquito larvae collections were conducted on different days in the same period to increase the chance of having offspring from different females. Larvae collection was performed at 3–4-day intervals for a total of 2 weeks to collect a sufficient quantity of mosquitoes for the bioassays. Larvae sampled from each breeding site were reared separately to the adult stage for molecular identification and insecticide susceptibility testing. Mosquito larvae were reared under standard insectary conditions (25–27 °C and 70–75% relative humidity) and fed daily with powdered Tetramin® baby fish food (Charterhouse Aquatics, London, UK) until their progression to the adult mosquito stage.

### PCR-based species identification

A total of 400 adult females of *Anopheles* (100 females from each vegetable farm) obtained from rearing were morphologically identified as belonging to *An. gambiae s.l*.^52^ These mosquitoes were subjected to molecular species-level identification, and 100 female mosquitoes from each farm were used. DNA extraction using the Livak protocol^53^ was first performed, and then SINE-PCR was carried out using the following primer set: forward-TCGCCTTAGACCTTGCGTTA and reverse-CGCTTCAAGAATTCGAGATAC as described by Santolamazza *et al*.^54^.

### Determination of insecticide residues in mosquito breeding habitats

Water samples containing soil sediments were collected from breeding sites using white glass cups, stored in properly labelled sterile glass bottles, covered with aluminium foil, kept in a cooler and transferred to the laboratory at IITA-Cotonou. Water samples were screened for λ-cyhalothrin residues. Pesticide residues (λ-cyhalothrin) were extracted from three replicates of each water sample (50 ml of water X3). The solid phase extraction (SPE) technique described by Guan and Meekin^55^ was used for separation, purification and preconcentration of λ-cyhalothrin residues in water samples before detection on an HPLC machine (Agilent Technologies 1260 infinity, Deutschland GmbH & Co. KG, Waldbronn, Germany). Briefly, the SPE solid sorbent (C18) was conditioned three times using 3 ml of methanol and 1 ml of sigma water followed by the percolation of 30 ml of the sample for analysis. The next step was washing of the solid sorbent with low-elution strength solvent to eliminate matrix components retained by the solid sorbent. The elution of λ-cyhalothrin residues was obtained using 3 ml of X3 acetonitrile. Subsequently, the extracted compounds were concentrated and dissolved in 1 ml of acetonitrile, and an aliquot of 75 μL from each sample was loaded in an HPLC system for quantification as described by Djouaka *et al*.^41^. A 5 μm, 120 Â, 4.6 × 250 mm C18 HPLC column was used (Thermo Scientific, USA), and the mobile phase (HPLC grade solutions, Sigma Aldrich) was a mixture of methanol and water (90:10). A flow rate of 1 mL/min was maintained, and the sample injection volume was 50 μL. The elution was monitored with an HPLC UV detector at 226 nm. The chromatographic peaks corresponding to the retention times in the column for each sample were identified and compared with the determined retention time of λ-cyhalothrin standard solutions. The λ-cyhalothrin concentration in each sample was later calculated using the equation generated from the standard curve.

### Screening for copper in surveyed *Anopheles* breeding sites

Fractions (70 ml) of prepared replicates of water samples used for insecticide residue analysis were also screened for the presence of copper. The analysis was performed using a Metalyser HM3000 system, which operates based on the voltammetric analysis technique (Trace2O, Berkshire, UK). Copper was analysed following the manufacturer’s instructions together with structured protocols (Trace2O, Berkshire, UK). Briefly, M4 reagents (buffers and standards) and working electrode 1 (WE1) were selected and used for the analysis. Electrode conditioning was first achieved with M1 4,5 solution and resulted in the formation of a thin grey plate on the surface. Then, one sachet of M1 4,5a and M1 4,5b buffers was added into the test beaker along with 70 ml of the sampled water before connecting it onto the analysis probe. After the first step of Cu screening, 280 μL of M4 standard, which corresponds to 20 ppb of Cu, was added into the preparation to reveal the concentration of Cu in the sample.

### Correlation analysis between the presence of xenobiotics in mosquito breeding habitats and insecticide resistance profiles of sampled mosquitoes

We conducted a correlation analysis between resistance profiles of sampled mosquitoes to λ-cyhalothrin and the contamination levels of their larval habitats. The purpose of this correlation analysis was to determine whether there is a link between breeding site contamination (copper and/or λ-cyhalothrin) and the selection of insecticide resistance in mosquito populations breeding in and around vegetable farms.

### Monitoring the mortality of *Anopheles* mosquitoes in simulated breeding sites contaminated with copper

#### Preparation of breeding sites and selection of Anopheles populations

The highest concentration (0.5601 ppm) of copper detected in the mosquito breeding water was used for the laboratory simulation experiments. Four strains of *Anopheles* mosquitoes were used in this laboratory assay: two wild strains of pyrethroid-resistant *Anopheles coluzzii;* one from the vegetable farm at Houeyiho (*An. coluzzii -Houeyiho*) and the second from an urban area in Ladji (*An. coluzzii-Ladji*), both localities found in Cotonou. Furthermore, two laboratory strains, *Anopheles gambiae Kisumu* (an insecticide-susceptible strain maintained in the laboratory for more than 20 years) and *Anopheles gambiae* VKPER (a laboratory strain that is resistant to permethrin and is selected from the Kou Valley in Burkina-Faso), were used. These *Anopheles* populations were reared separately in simulated breeding sites.

#### Experimental setup of simulated breeding sites and monitoring of mosquito mortalities

Four glass tubes, each containing 100 ml of deionised water contaminated with copper at 0.056 mg/L, were seeded with a total of 100 well-fed *Anopheles* larvae according to the standard WHO protocol^56^. Each glass tube had an average of 25 larvae (L2-L3 stages). We added 2 tubes of 25 larvae each seeded into deionised water alone (no copper contamination) to these 4 replicates, which served as control tubes, for a total of 150 larvae in each experimental block. Four experimental blocks were created to monitor the development of *An. coluzzii-Houeyiho*, *An. coluzzii-Ladji*, *An. gambiae* VKPER and *An. gambiae Kisumu* in the simulated breeding sites containing copper residues. Prior to this experiment, larvae were well fed overnight and then transferred into deionised water for one hour (observation time) before being seeded in solutions contained in glass tubes. Larvae were fed Tetramin® fish food daily and monitored until their emergence as adult mosquitoes. Larval mortality was then recorded at 24, 48, 72, 96, 120, 144 and 168 hours for both the experiment and control setups. The susceptibility/tolerance of mosquito larvae to copper was also analysed.

### Statistical analysis

Insecticide susceptibility test data were analysed using descriptive statistics and interpreted using the WHO guidelines^56^. Log probit analysis was used to examine the different mortality levels, while Abbott’s formula^57,58^ was used to correct mortalities higher than 20% in control samples.

Student’s t-test was used to analyse the KAP data using SPSS v 22.0 software. The correlation between the level of copper contamination and the insecticide resistance profile was analysed using linear regression. Additionally, linear regression was used to test for significant differences and correlations in mortalities between the susceptible *Anopheles gambiae Kisumu* strain and resistant mosquitoes under different exposure periods with GraphPad Prism 5. All data were tested at a significance level of P ≤ 0.05.

## Supplementary information

Supplementary information.
