# Supplementary material for: A preliminary analysis on the effect of copper on Anopheles coluzzii insecticide resistance in vegetable farms in Benin
Source: Sci Rep. 2020 Apr 14;10:6392. doi: 10.1038/s41598-020-63086-5 (PMC7156479; doi:10.1038/s41598-020-63086-5)
Supplement: Supplementary file 1 — Supplementary information. [file 41598_2020_63086_MOESM1_ESM.docx]

**‘A preliminary analysis on the effect of copper on *Anopheles coluzzii* insecticide resistance in vegetable farms in Benin’**

Armand Defo Talom^1,2^*, Michele Agnes Essoung^1,2^, Adam Gbankoto^3^, Genevieve Tchigossou^3,4^, Romaric Akoton^3,4^, Bio Bangana A. Sahabi^5^, Seun Michael Atoyebi^4,6^, Apollin Kuate Fotso^2^, Rudi L. Vespoor^7^; Manuele Tamò^4^, Timoleon Tchuinkam^1^, Gustave Leopold Lehman^8^, Jo Lines^9^, Charles S. Wondji^2,10,11^, Rousseau Djouaka^4^*

^1^University of Dschang, Vector Borne Diseases Laboratory (VBID) Po Box 067; ^2^International Institute of Tropical Agriculture, Yaoundé, Cameroon; ^3^University of Abomey Calavi, Laboratory of Experimental Physiology and Pharmacology, Faculty of Sciences and Technology BP 526 Cotonou, Benin; ^4^International Institute of Tropical Agriculture, Cotonou, 08 BP 0932, Benin; ^5^National University of Agriculture, Porto-Novo, Benin; ^6^Cell Biology and Genetics Unit, Department of Zoology, University of Ibadan, P.O. Box 5116, Oyo State, Nigeria; ^7^University of Liverpool, Institute of Integrative Biology, Liverpool, United Kingdom L697ZB; ^8^Faculty of Science University of Douala; ^9^London School of Hygiene & Tropical Medicine, London, UK; ^10^Liverpool School of Tropical Medicine, Pembroke Place, L3 5QA, Liverpool, UK; ^11^Centre for Research in Infectious Diseases (CRID),Cameroon;

*Corresponding authors

Rousseau Djouaka: r.djouaka@cgiar.org, +229 96804924

Armand Defo Talom: talomarmand@yahoo.fr, Tel: +237 675068181

**Supplementary file.**

**“Title: A preliminary analysis on the effect of copper on *Anopheles coluzzii* insecticide resistance in vegetable farms in Benin**”

**Questionnaire for vegetable farmers at Houeyiho and Seme**

**Country**……………………………………..….…… **Date and time**……………………………………………….………………………………….

**City**………………………………...…………….. **Name /first name of interviewer**………………………………..………………………..…

**District**………………………………………………....………. …….…………………...…………………………………………………………….

**Site** :…………………………………………………**Interviewed Ref number** :……………………..……………………………………………..

***Note :*** *Put a cross (×) for the right propositions.*

**1. Identification of farmers:**

**1.1. Name: ___**__________________________ **Surname: _______________________________________**

**1.2. Sex**: 1= Male___ 2=Female___

**1.3. Age:** _________ Years or born towards: __________________

**1.4. Marital status**: 1= Married ___ 2= Divorced (e)___ 3=Bachelor ___ 4= Widowed ___

**1.5. Household size: _______________**

**1.6. Main activity:** 1= Agriculture__ 2= Trading__ 3= Vegetable farming __ 4= Others_______________

**1.7. Since when have you started vegetable farming**? __________________________________

2. **General agricultural practices:**

**2.1. With who do you farm?**

1= My wife___ 2= My children ___ 3= My friends___ 4= Alone___ 5= Employee___- 6= Others__________________________________________________________________________________

**2.2. Which vegetable do you farm?**

1= Tomato __ 2= Carrot__ 3= Solanum macro carpon__ 4= Amaranthus __ 5= Cabbage__ 6= Aubergine__ 7= Vernonia__ 8= Ocimum gratissimum__ 9= Pepper__ 10= Cucumber __ 11= Lettuce__ 12= Green bean__ 13= Sweet pepper__ 14= Parsley__ 15= Others_______________________________________________________

**2.3. What are the 5 vegetables mostly sold? (Sorted by importance)**

(1) _______________ (2) ________________ (3) _______________ (4) _____________ (5) ____________

**2.4. Which type of water do you use for irrigation?**

1= Borehole water___ 2= Well___ 3= Swamp ___ 4= Basin__ 6=Others_________

**2.5. Which equipment do you use for irrigation?**

1= Watering can___ 2= Motor pump___ 3= Sprinkler___ 4= Links__ 5= Strip__ 6=Others__________

**2.6. Do you use fertilizers to grow vegetables?**

1= Yes___ 2= No ___

**2.7. If yes, which type of fertilizers do you use?**

1= Chemical fertilizers__ 2- organic fertilizers__ 3- Others (specified) ___________________________

**2.8. Which type of chemical fertilizers do you use?**

1= Urea___ 2=NPK__ 3= Others (specified)___________________________________________________

**2.9. Which type of organic fertilizers do you use?**

1= Dropping___ 2= Compost__ 3= Cow dung___ 4= Others (Specified)__________________

**2.10. Do you use pesticides for your vegetables?**

1= Yes___ 2= No ___

**2.11. If yes, which type of pesticides do you use?**

Fill the table below___________________________________________________________________

**2.12. Which protective equipment do you use during pesticides application?**

1= None__ 2= Gloves__ 3= labcoat__ 4=helmet__ 5= Special clothes__ 6=Others…………

Tableau 1: Identification des types de pesticides

| **Pesticide** | **Class** | **Vegetable** | **Application Period** | **Frequency** | **Quantity** |
| --- | --- | --- | --- | --- | --- |
|  | 1-2-3-4 |  | 1-In case of attack  2-Without attack  3-After 1 month  4- Others | 1- After 7 days  2- once/ 2 week  3- Three time/ month  4- Others |  |
|  | 1-2-3-4 |  | 1-In case of attack  2-Without attack  3-After 1 month  4- Others | 1- After 7 days  2- once/ 2 week  3- Three time/ month  4- Others |  |
|  | 1-2-3-4 |  | 1-In case of attack  2-Without attack  3-After 1 month  4- Others | 1- After 7 days  2- once/ 2 week  3- Three time/ month  4- Others |  |
|  | 1-2-3-4 |  | 1-In case of attack  2-Without attack  3-After 1 month  4- Others | 1- After 7 days  2- once/ 2 week  3- Three time/ month  4- Others |  |
|  | 1-2-3-4 |  | 1-In case of attack  2-Without attack  3-After 1 month  4- Others | 1- After 7 days  2- once/ 2 week  3- Three time/ month  4- Others |  |
|  | 1-2-3-4 |  | 1-In case of attack  2-Without attack  3-After 1 month  4- Others | 1- After 7 days  2- once/ 2 week  3- Three time/ month  4- Others |  |

**PESTICIDE CLASSES:** 1- Insecticides 2- herbicides 3- fungicides 4-Others (Specified)

**3. Perception of market gardeners on the quality of irrigation water**

**3.1. How do you find your irrigation water?**

1= Clean water___ 2= Wastewater___ 3= Others________________________________________

**3.2. In your opinion, what contaminant (s) do you think are present in the water?**

1 = Microorganisms_ 2 = Chemicals_3 = Heavy Metals_4 = Other _________________________

**3.3. What do you think is responsible for this contamination?**

1 = Fertilizers___ 2 = environment ___ 3 = faeces ___ 4 = storage tanks___ 5 = Insecticides___ 6 = smoke from vehicles or aircraft ___ 7 = Other__________________________

**3.4. Are you aware of hazards from contaminated irrigation water?**

1=Yes___ 2= No___

**3.5. If yes, which ones?**

1= Diseases ___ 2= Environmental pollution ___ 3= Mortality___ 4= Others____________________

**3.6. Who are the main victims?**

1= Vegetable farmers___ 2= Consumers___ 3= Others____________________________________________

**4. Perception on the quality of vegetables**

**4.1. Do you eat your produced vegetable?**

1- Yes__ 2-No__

**4.2. If yes, how often?**

**1=** regularly___ 2= Sometime___ 3= Never___

**4.3. Which cleaning techniques do you use for your vegetables?**

1-Tap/well water__ 2- Irrigation water__ 3- water+ bleach__ 4- Water+ vinegar__ 5- Cooking__ 6- Without cooking__ 7- Others (Specified)…………………………………………….……

**4.4. What are the diseases linked to the consumption of your products?**

1-Typhoid__ 2-Diarrhoea___, 3-stomach ach__ 4-Cholera__ 5-Vomitting__ 6-cough___ 7-Amoeba__ 8- Cancer__ 9- Nervous system disorders __ 10-Others_______________________________

**End of the QUESTIONNAIRE.**

**Thank you for your participation in this study.**
